# Polymorphisms in *MTNR1A* (rs2119882) and *CLOCK* (rs1801260) genes are associated with facial acne susceptibility in gas station workers

**DOI:** 10.1371/journal.pone.0329150

**Published:** 2025-07-24

**Authors:** Yi Chi, Xueqin Yang, Donglin Deng, Peimao Li, Yingbiao Zhang

**Affiliations:** 1 Department of Dermatology, Shenzhen Prevention and Treatment Center for Occupational Diseases, Shenzhen, Guangdong, China; 2 Pathology and Toxicology Institute, Shenzhen Prevention and Treatment Center for Occupational Diseases, Shenzhen, Guangdong, China; 3 Medical Laboratory, Shenzhen Prevention and Treatment Center for Occupational Diseases, Shenzhen, Guangdong, China; 4 Department of Occupational Health Surveillance, Shenzhen Prevention and Treatment Center for Occupational Diseases, Shenzhen, Guangdong, China; UCMI: University College MAIWP International, MALAYSIA

## Abstract

This study aimed to explore the relationship between circadian rhythm gene polymorphisms, specifically *MTNR1A* rs2119882 and *CLOCK* rs1801260, and the risk of acne in an occupational population. *MTNR1A* encodes a melatonin receptor involved in circadian rhythm regulation, while *CLOCK* is a core transcription factor in the molecular circadian clock. Both genes are essential in maintaining hormonal balance, sleep-wake cycles, and inflammatory responses—factors closely associated with acne pathogenesis. A case-control study was conducted among 90 participants, comprising acne-affected workers (AAG), acne-free workers (AFG), and healthy control group (HCG). Peripheral blood samples were collected, and DNA was extracted for genotyping of *MTNR1A* rs2119882 and *CLOCK* rs1801260 polymorphisms. Sociodemographic, lifestyle, and occupational data were obtained via structured interviews. Logistic regression models were used to assess the association between gene polymorphisms and acne risk, adjusting for relevant covariates. Sensitivity analyses were performed to evaluate the robustness of the findings. In the overall population, no significant association was found between *MTNR1A* rs2119882 polymorphisms and acne risk. However, *CLOCK* rs1801260 polymorphisms showed a strong association with acne susceptibility. Under the dominant model, participants carrying the AG/GG genotypes exhibited a significantly higher risk of developing acne compared to those with the AA genotype (unadjusted odds ratios (OR) = 3.79, 95% CI: 1.27–11.31; adjusted OR = 5.08, 95% CI: 1.41–18.33). In the additive model, the risk of acne increased with additional G alleles (unadjusted OR = 2.95, 95% CI: 1.22–7.13; adjusted OR = 3.51, 95% CI: 1.25–9.81). Subgroup analysis among night shift workers revealed a significant association between *MTNR1A* rs2119882 and acne risk, such that carriers of the CC genotype exhibited increased susceptibility (adjusted OR = 3.97, *p* = 0.049). Moreover, individuals with AG/GG genotypes at *CLOCK* rs1801260 showed an even higher risk (OR = 4.96, 95% CI: 1.22–20.14). This study suggests that circadian rhythm gene polymorphisms, particularly *CLOCK* rs1801260, are associated with acne risk, especially in individuals working rotating night shifts.

## Introduction

Acne is a widespread chronic inflammatory disease, ranked second among the skin diseases according to the Global Burden of Disease study [1], with a global prevalence of approximately 9.4% [2] predominantly seen in adolescents [1,3]. Acne affects 85% of adolescents and may persist beyond adolescent years into adulthood [4]. Genetic, environmental, and lifestyle factors are hypothesized to cause acne [5], such as excess sebum production by androgens, altered keratinization, propionibacterium acnes, inflammation, diet (high intake of sugary and fatty foods), sleep deprivation, and obstruction of the sebaceous follicles [5,6].

Circadian rhythm plays an important role in hormone synthesis, glucose and lipid metabolism, and chronic inflammation [7]. Disruption of circadian rhythms directly or indirectly influences a variety of physiological activities, including the sleep-wake cycle, hormone secretion, periodic changes in body temperature, and blood glucose regulation [8]. Shift work, including night shifts or rotating schedules, represents an irregular work pattern that may disrupt circadian rhythm [9,10]. Such circadian misalignment in night shift workers may contribute to the development of skin conditions, including acne [10].

Circadian-related genes, such as melatonin receptor genes (*MTNR*) and clock circadian regulator (*CLOCK*), participate in transcription-translation feedback loops that drive circadian oscillations, with expression levels cycling over a 24-hour period [11]. Melatonin, a major circadian hormone secreted by the pineal gland under the control of the suprachiasmatic nucleus (SCN), mediates its physiological effects primarily through membrane receptors *MTNR1A* and *MTNR1B* [12]. Among these, *MTNR1A* is more broadly expressed in the skin, including keratinocytes and dermal fibroblasts [13], and plays important roles in antioxidative and anti-inflammatory pathways [14,15]. The rs2119882 polymorphism, located in the promoter region of *MTNR1A*, is considered a key functional SNP, influencing *MTNR1A* expression [9,16,17].

Notably, *MTNR1A* appears to play a more direct role in acne pathogenesis compared to *MTNR1B.* It exhibits higher expression in skin and reproductive axis tissues [18], and is more closely linked to androgen-regulated sebum production and follicular keratinization—key features of acne [19–21]. Additionally, *MTNR1A* plays a stronger role in local anti-inflammatory responses, including suppression of IL-6 and IL-1β and modulation of macrophage activity [22,23]. Under stress conditions, it regulates the expression of steroidogenic genes and testosterone synthesis, and its promoter methylation is altered in inflammatory diseases and severe acne—underscoring its multifaceted role in acne pathogenesis [24–27]. These features collectively support the prioritization of *MTNR1A* in this study.

The *CLOCK* gene, located on chromosome 4q12 and comprising 20 exons, is a central component of the circadian system, regulating energy metabolism [28]. The rs1801260 polymorphism in *CLOCK*, located in the 3’ untranslated region (UTR), affects the binding of microRNAs and alters mRNA stability or translation efficiency [29]. It has been linked to metabolic syndrome [30], sleep disorders and obesity [31], which are known risk factors for acne development [32]. In human skin, *CLOCK* is rhythmically expressed and may modulate keratinocyte proliferation, lipid secretion, and cutaneous inflammation [33]. Circadian disruption due to night-shift work may be aggravated in individuals carrying specific *CLOCK* variants, a condition linked to impaired skin barrier repair and heightened inflammation—both relevant to acne [34].

In China, the overall prevalence of acne is approximately 8.1%, based on a large-scale, community-based epidemiological study involving 17,345 individuals across six cities, which found 1,399 confirmed cases of acne [35]. Occupational populations account for 50% to 70% of China’s total workforce, and Shenzhen hosts a substantial proportion of these workers. In 2018 alone, over 190,000 workers in Shenzhen were exposed to occupational hazards [36], which may increase their vulnerability to various health conditions, including acne [37].

The high prevalence of acne and its related complications in occupational populations— particularly those with irregular work schedules—underscores the importance of targeted investigations [31]. Circadian rhythm disruptions, often experienced by shift workers, have been strongly linked to sleep disorders, which are recognized as significant risk factors for acne [31]. Both genetic predisposition and environmental exposures (e.g., artificial light at night, volatile chemicals) may act synergistically in shift workers [38]. Polymorphisms in circadian genes, such as *MTNR1A* rs2119882 and *CLOCK* s1801260, may amplify the physiological effects of circadian misalignment, leading to increased sebum production, follicular inflammation, and oxidative stress—all hallmarks of acne development [29,31].

However, the direct role of these SNPs in acne susceptibility has not yet been investigated, particularly in environmentally stressed occupational groups such as gas station workers. This study aims to explore the relationship between *MTNR1A* rs2119882 and *CLOCK* rs1801260 variants and acne risk in Chinese occupational populations, with a focus on shift-working gas station employees. The findings are expected to provide crucial insights into the genetic predisposition to acne, guiding primary prevention strategies, screening of high-risk populations, and the development of targeted interventions for acne management.

## Materials and methods

### Study design and sample size

This case-control study aimed to investigate the association between genetic variations in *MTNR1A* and *CLOCK* genes (in terms of candidate single nucleotide polymorphisms, SNPs) and acne susceptibility. The study population consisted of fuel station workers and healthy individuals undergoing occupational health examinations at the Shenzhen Prevention and Treatment Center for Occupational Disease between July 2023 and July 2024.

The sample size was calculated using standard methods for case-control studies [39]:


$$\begin{array}{c} n=\frac{{\left(z_{\frac{\alpha }{2}}+z_{\beta }\right)}^{2}\times {(P}_{0}\times \left(1-P_{0}\right)+r\times P_{1}\times (1-P_{1}))}{r\times {\left(P_{1}-P_{0}\right)}^{2}} \end{array}$$ (1)

Assuming an exposure rate of 10% for the minor allele of *CLOCK* rs1801260 in the control group ($P_{0}$ = 0.10) [40], and 40% in the case group ($P_{1}\;$= 0.40), with a 1:2 case-control ratio (*r* = 1:2), α = 0.05, and 80% power (β=0.2), the required sample size was estimated at 78 (26 cases and 52 controls). To account for a 15% dropout rate, at least 30 cases and 60 controls were recruited in this study.

### Study subjects

A total of 30 fuel station workers with acne were randomly assigned to the acne-affected group (AAG), and 30 acne-free fuel station workers were selected for the acne-free group (AFG). Additionally, 30 healthy individuals undergoing routine health examinations during the same period were randomly selected as the healthy control group (HCG).

Inclusion criteria for study participants were as follows: (1) aged 18 years or older; (2) no history of hypertension, diabetes, cardiovascular disease, or malignancy, and no long-term use of medication for chronic diseases; (3) willingness to complete a structured questionnaire and undergo a facial skin examination; and (4) availability of peripheral blood samples for genetic analysis.

Acne is generally diagnosed through physical examination rather than laboratory tests [41]. Additionally, patient self-reporting has been validated as a reliable method in dermatology for identifying dermatological conditions and symptoms of acne [42]. In this study, acne diagnosis was based on the International Consensus on Acne Classification, which defines acne by the presence of comedones, papules, pustules, nodules, or cysts on the face and/or trunk as diagnostic criteria for acne [43]. Participants who did not exhibit any of these features were classified into the control group.

### Ethics

The study protocol was approved by the Ethics Committee of the Shenzhen Prevention and Treatment Center for Occupational Disease (Approval No. LL-2023019, approval date: June 29, 2023). All participants provided written informed consent before enrollment.

### DNA isolation and genotyping

Peripheral venous blood samples (5 mL) were collected from each participant into EDTA-K2 anticoagulant tubes by trained nurses during routine occupational health examinations. The tubes were gently inverted to ensure proper mixing with the anticoagulant and temporarily stored at −20°C. Subsequently, samples were then transported under cold chain conditions to the laboratory and stored at −80°C for future analyses.

Genomic DNA was extracted from whole blood using the Ezup column-based genomic DNA extraction kit (Sangon Biotech, China), according to the manufacturer’s protocols (https://www.sangon.com/productImage/DOC/B518253/B518253_ZH_P.pdf). Genotyping was performed for two SNPs associated with circadian regulation: *MTNR1A* gene rs2119882 and *CLOCK* gene rs1801260. The quality of DNA was assessed using a UV-Vis spectrophotometer (SMA4000; Merinton, China) to measure concentration and purity, and 1% agarose gel electrophoresis (AGE) (FR-980A; Shanghai Furi Technology Co., LTD, China) to evaluate DNA integrity [36].

Primer sequences for the *MTNR1A* rs2119882 and *CLOCK* rs1801260 (Table 1) were designed using Primer Premier 5 software. Polymerase chain reaction (PCR) amplification of *MTNR1A* and *CLOCK* SNPs was conducted under the following thermal cycling conditions: an initial denaturation at 95°C for 5 minutes; 10 cycles of denaturation at 94°C for 30 seconds, annealing starting at 63°C with a 0.5°C decrement per cycle for 30 seconds, and extension at 72°C for 30 seconds. This was followed by 30 cycles of denaturation at 95°C for 30 seconds, annealing at 58°C for 30 seconds, and extension at 72°C for 30 seconds. A final extension was performed at 72°C for 10 minutes to ensure complete elongation of PCR products. The amplified samples were then held at 4°C prior to downstream analysis.

**Table 1:** Primer sequences and characteristics for *MTNR1A* and *CLOCK* SNPs for the analysis.

Gene	SNP	Sequence (5’-3’)	L (bp)
***MTNR1A***	**rs2119882**	Forward: CCGTTCATTGTGTTTCCTCTCT	255
		Reverse: ATTATCGGAAACATCTAGCACCATC	
***CLOCK***	**rs1801260**	Forward: CCCTGGAGGTCATTTCATAGCT	230
		Reverse: AAGTTCCAGCAGTTTCATGAGAT	

SNP, single nucleotide polymorphism; L, length of amplicons.

A 5 μl aliquot of the PCR product was analyzed via 1% AGE to assess the presence and integrity of the amplified DNA. The target PCR bands were isolated and purified using the SanPrep Column DNA Gel Extraction Kit (Sangon Biotech, China), following the manufacturer’s protocol. Genotyping of the SNPs rs2119882 and rs1801260 was performed using the 3730XL DNA Analyzer (Applied Biosystems, USA). The same primer pairs used in the PCR amplification were also used for bidirectional Sanger sequencing of the amplicons. The resulting data were processed and analyzed using sequence analysis software to confirm the SNP genotypes. As a quality control measure, 5% of randomly selected samples were genotyped in blinded duplicates, and the results were 100% concordant.

### Covariate assessment

A standardized questionnaire was administered via face-to-face interviews by trained medical staff to collect potential risk factors for acne [44,45]. The questionnaire encompassed three domains: (1) sociodemographic data, including age, gender, height, weight, marital status, education level, skin type, and family history of acne; (2) lifestyle factors, such as smoking and alcohol consumption, physical activity frequency, daily water intake, and dietary preferences; and (3) occupational factors, including rotating night shift work and sleep duration.

Smoking was defined as having smoked at least one cigarette per day for a minimum of one year, while alcohol consumption was defined as drinking 50 grams of wine or one bottle of beer per day for at least one year [46]. Individuals who had alternated between day and night shifts for more than one year and were still working rotating night shifts at the time of the survey were classified as “night shift workers” [47].

### Statistical analysis

The Shapiro–Wilk test was used to assess the normality of continuous variables, which were presented as mean ± standard deviation (SD) for normally distributed data. Group comparisons were performed using one-way analysis of variance (ANOVA) for continuous variables, while categorical variables were summarized as frequencies and percentages and compared using the Chi-square test. The Hardy-Weinberg equilibrium (HWE) for each SNP was also tested using the Chi-square test.

Univariate logistic regression was used to explore the associations between *MTNR1A* and *CLOCK* polymorphisms and acne risk. Multivariate logistic regression was then conducted to adjust for potential confounders, with results expressed as crude and adjusted odds ratios (ORs) along with 95% confidence intervals (CIs). Variables with a *p*-value < 0.100 in univariate analysis were included in the multivariate model to avoid excluding potentially important predictors [48]. This threshold was used solely for variable inclusion and not for determining statistical significance.

Genetic associations were further examined using five inheritance models: codominant, dominant, recessive, overdominant, and additive. In the codominant model, each genotype (e.g., TT, TC, CC) was analyzed as a separate category, with the major homozygous genotype used as the reference. The dominant model compared individuals carrying at least one minor allele (e.g., TC + CC) against major allele homozygotes (e.g., TT). The recessive model compared minor allele homozygotes (e.g., CC) to the combined group of heterozygotes and major homozygotes (TT + TC). In the overdominant model, heterozygotes (e.g., TC) were compared to both homozygous groups (TT + CC). The additive model assumed a linear effect of allele dosage, coded as 0, 1, or 2, corresponding to the number of minor alleles carried. To estimate the ORs and 95% CI, both univariate and multivariate logistic regression analyses were applied to each genetic model. This approach is consistent with established practices in genetic epidemiology [48].

To evaluate the robustness of these associations, additional stratified univariate and multivariate logistic regression analyses were performed within the fuel station population. Sensitivity analyses were also conducted using two alternative reference groups—healthy individuals and acne-free fuel station workers—to assess the stability of the gene-acne associations across different reference populations.

Statistical significance was set at *p-*value < 0.05 and all analyses were performed with R software (version 4.3.3).

## Results

### Comparison of characteristics among the three groups

A total of 90 participants, aged 18–46 years (mean age: 27.52 ± 6.67 years), were included in the study, of whom 77 (85.56%) were male. The proportion of male participants was higher in the HCG (100%) and AFG (83.33%) groups compared to the AAG group (73.33%). Additionally, participants in the AAG group had the shortest average sleep duration, and 93.33% of them involved in rotating night shift work (*p* < 0.05). No statistically significant differences were observed in variables such as family history of acne, skin type, or dietary habits (*p* > 0.05), as shown in Table 2.

**Table 2:** Comparison of characteristics between acne-affected and control groups.

Variables	Total (*n* = 90)	HCG (*n* = 30)	AFG (*n* = 30)	AAG (*n* = 30)	*p*-value
**Age, years**	27.52 ± 6.67	28.97 ± 6.95	27.87 ± 7.10	25.73 ± 5.68	0.162
**BMI, kg/m** ^**2**^	22.04 ± 2.71	22.12 ± 2.60	22.08 ± 3.00	21.93 ± 2.59	0.961
**Sex, *n* (%)**					0.007
** Male**	77 (85.56)	30 (100.00)	25 (83.33)	22 (73.33)	
** Female**	13 (14.44)	0 (0.00)	5 (16.67)	8 (26.67)	
**Marital status, *n* (%)**					0.565
** Single**	5 (5.56)	3 (10.00)	0 (0.00)	2 (6.67)	
** Married**	78 (86.67)	25 (83.33)	27 (90.00)	26 (86.67)	
** Divorced/Widowed**	7 (7.78)	2 (6.67)	3 (10.00)	2 (6.67)	
**Education, *n* (%)**					0.231
** Middle school or below**	16 (17.78)	3 (10.00)	5 (16.67)	8 (26.67)	
** High school**	59 (65.56)	21 (70.00)	18 (60.00)	20 (66.67)	
** University or above**	15 (16.67)	6 (20.00)	7 (23.33)	2 (6.67)	
**Sleep duration (Hours)**	6.62 ± 1.22	6.97 ± 1.19	6.70 ± 1.26	6.20 ± 1.13	0.046
**Skin types, *n* (%)**					0.691
** Oily skin**	42 (46.67)	12 (40.00)	12 (40.00)	18 (60.00)	
** Dry skin**	11 (12.22)	4 (13.33)	5 (16.67)	2 (6.67)	
** Normal skin**	10 (11.11)	4 (13.33)	4 (13.33)	2 (6.67)	
** Combination skin**	27 (30.00)	10 (33.33)	9 (30.00)	8 (26.67)	
**Family history of acne, *n* (%)**					0.201
** No**	35 (38.89)	12 (40.00)	15 (50.00)	8 (26.67)	
** Yes**	55 (61.11)	18 (60.00)	15 (50.00)	22 (73.33)	
**Smoking status, *n* (%)**					0.782
** No**	61 (67.78)	19 (63.33)	22 (73.33)	20 (66.67)	
** Yes**	29 (32.22)	11 (36.67)	8 (26.67)	10 (33.33)	
**Drinking status, *n* (%)**					0.734
** No**	70 (77.78)	25 (83.33)	22 (73.33)	23 (76.67)	
** Yes**	20 (22.22)	5 (16.67)	8 (26.67)	7 (23.33)	
**Rotating night shift work, *n* (%)**					0.009
** No**	20 (22.22)	12 (40.00)	6 (20.00)	2 (6.67)	
** Yes**	70 (77.78)	18 (60.00)	24 (80.00)	28 (93.33)	
**Physical activity frequency, *n* (%)**					0.956
** No**	35 (38.89)	12 (40.00)	10 (33.33)	13 (43.33)	
** 1-2 times per week**	33 (36.67)	11 (36.67)	12 (40.00)	10 (33.33)	
** ≥3 times per week**	22 (24.44)	7 (23.33)	8 (26.67)	7 (23.33)	
**Water intake per day, *n* (%)**					0.061
** ≤ 600 mL**	24 (26.67)	5 (16.67)	8 (26.67)	11 (36.67)	
** 601-1500 mL**	44 (48.89)	12 (40.00)	17 (56.67)	15 (50.00)	
** >1500 mL**	22 (24.44)	13 (43.33)	5 (16.67)	4 (13.33)	
**Dietary habits, *n* (%)**					0.800
** Spicy food**	13 (14.44)	5 (16.67)	5 (16.67)	3 (10.00)	
** Sweet food**	25 (27.78)	7 (23.33)	8 (26.67)	10 (33.33)	
** Fatty food**	33 (36.67)	10 (33.33)	10 (33.33)	13 (43.33)	
** Light food**	19 (21.11)	8 (26.67)	7 (23.33)	4 (13.33)	

BMI, body mass index; HCG, healthy control group; AFG, acne-free group; AAG, acne-affected group.

### The fragment of PCR amplification

**Fig 1:**
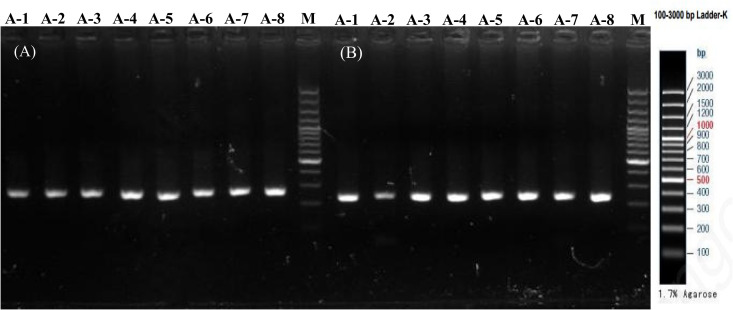
The electrophoresis image of fragments of PCR amplification for rs2119882 and rs1801260. Agarose gel electrophoresis was used to assess the quality and specificity of primers for **(A)** *MTNR1A* rs2119882 and **(B)** *CLOCK* rs1801260. Lanes A-1 to A-8 represent DNA samples from acne-free gas station workers (AFG group).

The PCR amplification fragments for the *MTNR1A* and *CLOCK* gene SNPs (rs2119882 and rs1801260) are displayed in Fig 1. The electrophoresis results demonstrated that the amplified products yielded single, well-defined bands at the expected positions, with a clear background, indicating successful amplification.

### HWE analysis for MTNR1A and CLOCK genes

At the *MTNR1A* rs2119882 locus, three genotypes (TT, TC, and CC) were detected, while the *CLOCK* rs1801260 locus exhibited the AA, AG, and GG genotypes, as shown in S1 Fig. The most common alleles were rs2119882-T and rs1801260-A, present in 57.22% and 88.33% of participants, respectively. HWE testing confirmed that both the *MTNR1A* rs2119882 and *CLOCK* rs1801260 loci were in equilibrium across all three groups, suggesting the representativeness and genetic stability of the study population (*p* > 0.05, S1 Table).

### Association between *MTNR1A* and *CLOCK* gene polymorphisms and acne risk

Table 3 presents the genotype distributions of *MTNR1A* rs2119882 (TT, TC, CC) and *CLOCK* rs1801260 (AA, AG, GG) under codominant, dominant, recessive, and overdominant genetic inheritance models. No statistically significant differences in genotype frequencies were observed among the HCG, AFG, and AAG groups (*p*-value > 0.05, S2 Table).

**Table 3:** Association between *MTNR1A* and *CLOCK* genes polymorphisms and acne risk among night shift workers.

*Gene*	Gene model	Genotype	Crude Model		Adjusted Model	
			OR (95% CI)	*p*-value	OR (95% CI)	*p*-value
***MTNR1A* gene rs2119882 locus**	**Codominant**	**TT**	*Ref*		*Ref*	
		**TC**	1.93 (0.59–6.26)	0.276	2.29 (0.61–8.61)	0.220
		**CC**	3.00 (0.79–11.42)	0.107	**3.97 (1.01–15.69)**	**0.049**
	**Dominant**	**TT**	*Ref*		*Ref*	
		**TC + CC**	2.26 (0.75–6.76)	0.146	2.81 (0.82–9.61)	0.100
	**Recessive**	**TT + TC**	*Ref*		*Ref*	
		**CC**	2.01 (0.67–6.08)	0.215	2.41 (0.71–8.13)	0.157
	**Overdominant**	**TT + CC**	*Ref*		*Ref*	
		**TC**	1.16 (0.44–3.02)	0.768	1.19 (0.41–3.46)	0.747
	**Additive**	**–**	1.73 (0.89–3.37)	0.105	**1.99 (1.01–3.93)**	**0.047**
***CLOCK* gene rs1801260 locus**	**Codominant**	**AA**	*Ref*		*Ref*	
		**AG**	2.92 (0.73–11.62)	0.128	4.08 (0.85–19.53)	0.079
		**GG**	5.84 (0.57–60.04)	0.138	8.69 (0.66–115.04)	0.101
	**Dominant**	**AA**	*Ref*		*Ref*	
		**AG + GG**	**3.51 (1.03–11.94)**	**0.045**	**4.96 (1.22–20.14)**	**0.025**
	**Recessive**	**AA + AG**	*Ref*		*Ref*	
		**GG**	4.92 (0.48–49.93)	0.178	6.69 (0.51–87.16)	0.147
	**Overdominant**	**AA + GG**	*Ref*		*Ref*	
		**AG**	2.59 (0.66–10.19)	0.173	3.37 (0.73–15.61)	0.120
	**Additive**	**–**	**2.62 (1.01–6.77)**	**0.047**	**3.37 (1.14–9.95)**	**0.028**

Ref, reference category; OR, odds ratio; 95% CI, 95% confidence interval; Crude Model, univariate analysis; Adjusted Model, adjusted for potential confounders with a univariate analysis *p* < 0.100; bold values, it indicates that it is statistically significant.

**Fig 2:**
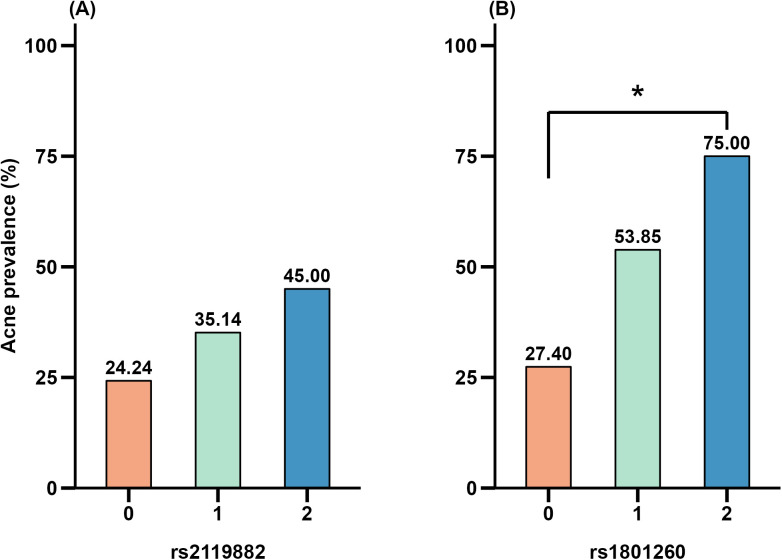
Association between acne risk and *MTNR1A* and *CLOCK* gene polymorphisms. (A) was *MTNR1A* rs2119882; (B) was *CLOCK* rs1801260. Each bar represents the proportion of acne cases within each genotype group. The exact acne prevalence (%) is labeled above each bar. Asterisks (*) indicate statistically significant differences (**p* *< 0.05).

However, when analyzing the distribution of acne cases across different genotypes of the *MTNR1A* rs2119882 and *CLOCK* rs1801260 polymorphisms, individuals with the GG genotype exhibited a significantly higher acne prevalence (75%) compared to those with the AA genotype (27.4%) (*p* = 0.034; Fig 2B). This suggests a potential association between the GG genotype of *CLOCK* rs1801260 and increased acne susceptibility, as shown in Fig 2.

### CLOCK rs1801260 polymorphisms increase acne risk

To evaluate the association between gene polymorphisms and acne risk, the HCG and AFG groups were combined as a unified control group, while the AAG group was designated as the case group. Binary logistic regression was used to explore the associations of different genetic models of *MTNR1A* rs2119882 and *CLOCK* rs1801260 with acne susceptibility. The multivariate model was adjusted for potential confounders identified in the univariate analysis (*p* < 0.100).

**Fig 3:**
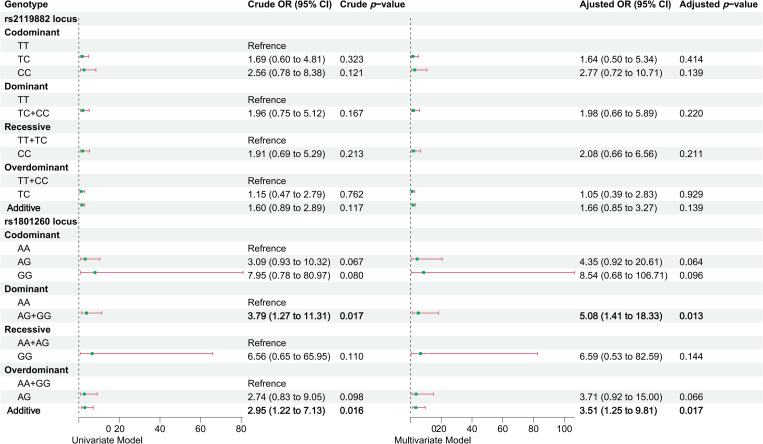
Association analysis of *MTNR1A* rs2119882 and *CLOCK* rs1801260 polymorphisms with acne risk under five genetic models. Odds ratios (ORs) were calculated using logistic regression, with reference genotypes defined by the major allele homozygotes. For the additive model, genotypes were coded based on the number of minor alleles (0, 1, 2). Error bars represent 95% confidence intervals. Bolded values indicate statistically significant results (*p* < 0.05).

The results indicated a significant allelic association at the *CLOCK* rs1801260 locus. Univariate and multivariate logistic regression analyses under the dominant model (AG + GG vs AA) revealed that workers carrying the AG/GG genotypes had a markedly higher risk of developing acne compared to those with the AA genotype (unadjusted OR = 3.79, 95% CI: 1.27–11.31; adjusted OR = 5.08, 95% CI: 1.41–18.33). Under the additive model (per G allele), acne risk increased progressively with each additional G allele (unadjusted OR = 2.95, 95% CI: 1.22–7.13; adjusted OR = 3.51, 95% CI: 1.25–9.81), as shown in Fig 3.

### Subgroup analysis in rotating night shift workers

Due to the limited number of non-night shift workers (*n* = 20, with only 2 cases of acne), subgroup analysis was restricted to participants engaged in rotating night shifts. The multivariate model revealed that the rs2119882 variant of the *MTNR1A* gene in homozygosis (CC) was most strongly associated with acne risk (OR = 3.97, 95% CI: 1.01–15.69; *p* = 0.049). Under the additive model, the risk of acne increased with each additional C allele (OR = 1.99, 95% CI: 1.01–3.93). Similarly, for the *CLOCK* rs1801260 variant, carriers of the minor genotype (AG/GG) showed significant associations with acne among rotating night shift workers under both dominant and additive models (Table 3).

### Sensitivity analysis

The sensitivity analysis revealed that, when using the HCG group as the control, the homozygous AA genotype of *CLOCK* rs1801260 was associated with the lowest acne risk, consistent with the results presented in Fig 3. However, when the AFG group was used as the control, a significant association was observed only in the multivariate analysis under the dominant model, where workers carrying the AG/GG genotypes had a higher risk of acne compared to those with the AA genotype. No significant association was identified under the additive model, as detailed in S3 and S4 Tables.

## Discussion

This case-control study presents a novel exploration of the relationship between circadian rhythm gene polymorphisms and acne risk in an occupational population. While *MTNR1A* rs2119882 was not significantly associated with acne susceptibility in the overall population, night shift workers carrying the CC genotype showed a markedly increased risk of (adjusted OR = 3.97, 95% CI: 1.01–15.69) and the C allele remained a risk factor under the additive model. In contrast, the *CLOCK* rs1801260 G allele was consistently associated with increased acne susceptibility across models, with higher risk observed in night shift workers carrying AA/AG genotypes. These findings highlight the influence of circadian gene variants, particularly in the context of circadian disruption, may contribute to pathogenesis through gene-environment interactions.

*MTNR1A*, a key G protein-coupled receptor, mediates melatonin’s physiological effects and is involved in steroid hormone synthesis, metabolic regulation, and the apoptosis of granulosa cells [49]. It also regulates the antioxidative and anti-inflammatory function of melatonin, which are crucial for maintaining skin health [50]. The rs2119882 polymorphism, located in the promoter region of the *MTNR1A* gene, may influence gene transcription by altering transcription factor binding, thereby modulating receptor expression.

Although the CC genotype of *MTNR1A* rs2119882 did not reach statistical significance in the overall population, the observed trend toward increased acne risk aligns with findings in the night shift worker subgroup, where a significant association was detected (adjusted OR = 3.97, *p* = 0.049). This suggests that limited sample size in the general analysis may have underpowered detection of a real effect. Additionally, a similar upward trend in acne prevalence among C allele carriers was observed in the night shift subgroup, indicating a potential biological effect that warrants further validation in larger cohorts.

Rotating night shift workers are particularly vulnerable to circadian rhythm disruption, often due to prolonged exposure to artificial light at night. Blue-enriched and high-intensity light can suppress melatonin release, especially when exposure occurs during biologically sensitive windows [51,52]. In individuals with the C allele, potential downregulation of *MTNR1A* expression may further compromise melatonin signaling, diminishing its protective effects against oxidative stress and inflammation [53]. However, it is also important to note that appropriately timed exposure to light—particularly in the early biological night—can support circadian entrainment in night shift workers, potentially mitigating melatonin suppression and improving physiological adaptation.

This gene–environment interaction may promote a proinflammatory cutaneous microenvironment, thereby increasing acne susceptibility. *MTNR1A* has previously been implicated in several inflammatory and endocrine-related disorders, such as polycystic ovary syndrome [54] and sleep disorders [55], both of which share mechanistic pathways with acne, including hormonal imbalance and immune dysregulation. Given the overlap, it is biologically plausible that dysfunction of *MTNR1A* under circadian disruption contributes to acne pathogenesis. These findings highlight the importance of integrating both genetic predisposition and environmental light exposure in acne risk evaluation.

Our findings revealed a robust association between the *CLOCK* rs1801260 polymorphism and acne susceptibility. Under the dominant model, individuals carrying the AG/ GG genotypes exhibited a significantly higher risk of developing acne compared to those with the AA genotype (OR = 5.08, 95% CI = 1.41–18.33). This risk was further elevated among rotating night shift workers (OR = 4.96, 95% CI = 1.22–20.14). Sensitivity analyses confirmed the consistency of this association, indicating the GG genotype may amplify acne risk especially in populations vulnerable to circadian disruption. Previous studies have established a link between the rs1801260 variant and sleep disorders [32,56,57], which are recognized contributors to acne development. Moreover, *CLOCK* gene variants are known to influence sleep quality and circadian regulation [58], reinforcing the biological plausibility of our findings.

The *CLOCK* gene, as a central regulator of circadian rhythms, coordinates key biological processes including skin barrier maintenance, inflammation control, and cellular repair [53]. The rs1801260 polymorphism, located in the 3′UTR of the *CLOCK* gene, may disrupt microRNA binding, thereby altering post-transcriptional regulation and affecting mRNA stability or translation efficiency [29]. The G allele, present in AG and GG genotypes, is thought to reduce miRNA binding affinity (particularly for miR-182 and miR-107), leading to decreased *CLOCK* protein expression. This downregulation may weaken the molecular circadian feedback loop and impair the rhythmic expression of *CLOCK*-regulated targets such as PPARα and Rev-Erbα, which are crucial for controlling lipid metabolism, sebocyte activity, and cutaneous inflammation [59].

*CLOCK* also influences insulin sensitivity and energy balance through its interaction with Sirt1, a key NAD + -dependent deacetylase that modulates fat and glucose metabolism [60,61]. Polymorphisms in *CLOCK* and other core circadian genes have been associated with metabolic syndrome, a condition strongly associated with acne pathogenesis [62]. In individuals carrying the AG or GG genotypes, particularly under circadian disruption such as night shifts or artificial light exposure, these molecular disturbances may promote excessive sebum production, increased inflammatory cytokine expression (e.g.,: IL-1β, IL-17, IL-6, TNF-α, IFN-γ) [63], and skin barrier dysfunction, ultimately contributing to acne susceptibility [63,64].

In modern society, circadian rhythm disruption is increasingly common due to factors such as night shifts, rotating work schedules, and trans-meridian travel [47]. Such disruption is often exacerbated by continuous exposure to artificial light at night, which induces neuroendocrine stress, particularly in shift workers. Prolonged stress leads to the activation of the hypothalamic-pituitary-adrenal (HPA) axis [12]. This process initiates the secretion of corticotropin-releasing hormone (CRH), followed by adrenocorticotrophic hormone (ACTH), which stimulates the adrenal cortex to release glucocorticoids [65,66]. Glucocorticoids, crucial in metabolic regulation, also affect circadian rhythms with peak secretion early in the morning [67]. Disrupted circadian alignment, as often seen in shift workers, can elevate glucocorticoid levels and exacerbate stress responses, thereby potentially increasing acne susceptibility [64,68].

In the sensitivity analysis, the study confirmed the robustness of the rs1801260 association, particularly among night shift workers, who are more prone to circadian rhythm disruptions. These findings support a gene–environment interaction model, in which the rs1801260 G allele, under conditions of circadian disruption, may exacerbate metabolic and inflammatory dysregulation, thereby increasing susceptibility to acne.

This study has several limitations that must be considered. The relatively small sample size (*n* = 90) may reduce the statistical power and limit the generalizability of the findings. Moreover, the study population was limited to gas station workers engaged in rotating night shifts, which may not fully be representative of the general population, particularly those without circadian rhythm disruption. The cross-sectional design precludes any inference of causality between gene polymorphisms and acne susceptibility. Although adjustments were made for confounders such as age and family history, residual confounding may persist due to unmeasured variables like diet, skincare habits, and workplace chemical exposures. Furthermore, the absence of direct circadian biomarkers, such as melatonin levels, limits the ability to definitively link between *CLOCK* gene variation and acne pathogenesis. Further studies with larger, more diverse cohorts and longitudinal designs are warranted to confirm and extend these findings.

## Conclusions

Our findings suggest that the *CLOCK* rs1801260 polymorphism, particularly the GG genotype, may increase acne susceptibility—like through circadian rhythm disruption and its downstream effects on metabolic and inflammatory pathways, particularly in shift-working populations. Future research is needed to elucidate the mechanistic links between circadian genes variants and dermatological disorders such as acne, and to evaluate circadian-targeted strategies for prevention and treatment.

## Supporting information

S1 Table HWE assumption test for all the SNPs analyzed in this study among three groups. (DOCX)

S2 Table Genotype and allele frequency distributions of *TNR1A* and *CLOCK* polymorphisms compared among HCG, AFG, and AAG groups. (DOCX)

S3 Table Case-control analysis of the association between *MTNR1A* and *CLOCK* gene polymorphisms and acne risk using the HCG as a control. (DOCX)

S4 Table Case-control analysis of the association between *MTNR1A* and *CLOCK* gene polymorphisms and acne risk using the AFG as a control. (DOCX)

S1 Fig Gene sequencing map (A) *MTNR1A* rs2119882 and (B) *CLOCK* rs1801260. (TIF)

S1 Checklist PLOS One human subjects research checklist. (DOCX)
