# Supplementary material for: Polymorphisms in MTNR1A (rs2119882) and CLOCK (rs1801260) genes are associated with facial acne susceptibility in gas station workers
Source: PLoS One. 2025 Jul 24;20(7):e0329150. doi: 10.1371/journal.pone.0329150 (PMC12289049; doi:10.1371/journal.pone.0329150)
Supplement: S1 Table — (DOCX) [file pone.0329150.s001.docx]

**S1 Table. HWE assumption test for all the SNPs analyzed in this study among three groups.**

| ***Gene*** | **SNP ID** | **Genotype** | **HCG** | | | | **AFG** | | | | **AAG** | | | |
| --- | --- | --- | --- | --- | --- | --- | --- | --- | --- | --- | --- | --- | --- | --- |
|  |  |  | **Observed** | **Expected** | $\boldsymbol{x}^{\boldsymbol{2}}$ | ***p*-value** | **Observed** | **Expected** | $\boldsymbol{x}^{\boldsymbol{2}}$ | ***p*-value** | **Observed** | **Expected** | $\boldsymbol{x}^{\boldsymbol{2}}$ | ***p*-value** |
| ***MTNR1A*** | **rs2119882** | **TT** | 14 | 13.33 | 0.300 | 0.693 | 11 | 9.63 | 1.033 | 0.461 | 8 | 7.01 | 0.526 | 0. 469 |
|  |  | **TC** | 12 | 13.34 |  |  | 12 | 14.74 |  |  | 13 | 14.98 |  |  |
|  |  | **CC** | 4 | 3.33 |  |  | 7 | 5.63 |  |  | 9 | 8.01 |  |  |
| ***CLOCK*** | **rs1801260** | **AA** | 27 | 27.08 | 0.083 | 1.00. | 26 | 25.21 | 3.580 | 0.130 | 20 | 18.41 | 2.932 | 0.081 |
|  |  | **AG** | 3 | 2.86 |  |  | 3 | 4.58 |  |  | 7 | 10.18 |  |  |
|  |  | **GG** | 0 | 0.07 |  |  | 1 | 0.21 |  |  | 3 | 1.41 |  |  |

HWE, Hardy-Weinberg equilibrium; HCG, healthy control group; AFG, acne-free group; AAG, acne-affected group.
