# Supplementary material for: Polymorphisms in MTNR1A (rs2119882) and CLOCK (rs1801260) genes are associated with facial acne susceptibility in gas station workers
Source: PLoS One. 2025 Jul 24;20(7):e0329150. doi: 10.1371/journal.pone.0329150 (PMC12289049; doi:10.1371/journal.pone.0329150)
Supplement: S2 Table — (DOCX) [file pone.0329150.s002.docx]

**S2 Table*.* Genotype and allele frequency distributions of *TNR1A* and *CLOCK* polymorphisms compared among HCG, AFG, and AAG groups.**

| **Variables** | **Gene model** | **Genotype** | **HCG**  **(*n* = 30)** | **AFG**  **(*n* = 30)** | **AAG**  **(*n* = 30)** | ***p*-value** |
| --- | --- | --- | --- | --- | --- | --- |
| ***MTNR1A* gene rs2119882 locus** | **Codominant** | TT | 14 (46.67) | 11 (36.67) | 8 (26.67) | 0.472 |
|  |  | TC | 12 (40.00) | 12 (40.00) | 13 (43.33) |  |
|  |  | CC | 4 (13.33) | 7 (23.33) | 9 (30.00) |  |
|  | **Dominant** | TT | 14 (46.67) | 11 (36.67) | 8 (26.67) | 0.299 |
|  |  | TC+CC | 16 (53.33) | 19 (63.33) | 22 (73.33) |  |
|  | **Recessive** | TT+TC | 26 (86.67) | 23 (76.67) | 21 (70.00) | 0.336 |
|  |  | CC | 4 (13.33) | 7 (23.33) | 9 (30.00) |  |
|  | **Overdominant** | TT+CC | 18 (60.00) | 18 (60.00) | 17 (56.67) | 1.00 |
|  |  | TC | 12 (40.00) | 12 (40.00) | 13 (43.33) |  |
| ***CLOCK* gene rs1801260 locus** | **Codominant** | AA | 27 (90.00) | 26 (86.67) | 20 (66.67) | 0.135 |
|  |  | AG | 3 (10.00) | 3 (10.00) | 7 (23.33) |  |
|  |  | GG | 0 (0.00) | 1 (3.33) | 3 (10.00) |  |
|  | **Dominant** | AA | 27 (90.00) | 26 (86.67) | 20 (66.67) | 0.057 |
|  |  | AG+GG | 3 (10.00) | 4 (13.33) | 10 (33.33) |  |
|  | **Recessive** | AA+AG | 30 (100.00) | 29 (96.67) | 27 (90.00) | 0.318 |
|  |  | GG | 0 (0.00) | 1 (3.33) | 3 (10.00) |  |
|  | **Overdominant** | AA+GG | 27 (90.00) | 27 (90.00) | 23 (76.67) | 0.279 |
|  |  | AG | 3 (10.00) | 3 (10.00) | 7 (23.33) |  |

HCG, healthy control group; AFG, acne-free group; AAG, acne-affected group.
