# Supplementary material for: Polymorphisms in MTNR1A (rs2119882) and CLOCK (rs1801260) genes are associated with facial acne susceptibility in gas station workers
Source: PLoS One. 2025 Jul 24;20(7):e0329150. doi: 10.1371/journal.pone.0329150 (PMC12289049; doi:10.1371/journal.pone.0329150)
Supplement: S3 Table — (DOCX) [file pone.0329150.s003.docx]

**S3 Table*.* Case-control analysis of the association between *MTNR1A* and *CLOCK* gene polymorphisms and acne risk using the HCG as a control.**

| ***Gene*** | **Gene model** | **Genotype** | **Crude Model** | | **Adjusted Model** | |
| --- | --- | --- | --- | --- | --- | --- |
|  |  |  | ***OR* (95% *CI*)** | ***p*-value** | ***OR* (95% *CI*)** | ***p*-value** |
| ***MTNR1A* gene rs2119882 locus** | **Codominant** | **TT** | *Ref* |  | *Ref* |  |
|  |  | **TC** | 1.90 (0.59–6.11) | 0.284 | 1.97 (0.41–9.35) | 0.396 |
|  |  | **CC** | 3.94 (0.91–17.01) | 0.066 | 4.89 (0.79–30.23) | 0.088 |
|  | **Dominant** | **TT** | *Ref* |  | *Ref* |  |
|  |  | **TC+CC** | 2.41 (0.82–7.10) | 0.111 | 2.73 (0.65–11.48) | 0.171 |
|  | **Recessive** | **TT+TC** | *Ref* |  | *Ref* |  |
|  |  | **CC** | 2.79 (0.75–10.33) | 0.126 | 3.31 (0.7–15.7) | 0.131 |
|  | **Overdominant** | **TT+CC** | *Ref* |  | *Ref* |  |
|  |  | **TC** | 1.15 (0.41–3.2) | 0.793 | 0.99 (0.27–3.65) | 0.987 |
|  | **Additive** | **-** | 1.97 (0.96–4.03) | 0.063 | 2.2 (0.89–5.45) | 0.089 |
| ***CLOCK* gene rs1801260 locus** | **Codominant** | **AA** | *Ref* |  | *Ref* |  |
|  |  | **AG** | 3.15 (0.72–13.71) | 0.126 | 4.42 (0.69–28.43) | 0.117 |
|  |  | **GG** | 21129337.07 (0–Inf) | 0.990 | 109809641.7 (0–Inf) | 0.995 |
|  | **Dominant** | **AA** | *Ref* |  | *Ref* |  |
|  |  | **AG+GG** | **4.50 (1.09–18.5)** | **0.037** | **5.94 (1.01–34.82)** | **0.048** |
|  | **Recessive** | **AA+AG** | *Ref* |  | *Ref* |  |
|  |  | **GG** | 17390400.88 (0–Inf) | 0.990 | 80433719.16 (0–Inf) | 0.995 |
|  | **Overdominant** | **AA+GG** | *Ref* |  | *Ref* |  |
|  |  | **AG** | 2.74 (0.63–11.82) | 0.177 | 3.71 (0.6–22.86) | 0.157 |
|  | **Additive** | **-** | **4.05 (1.13–14.55)** | **0.032** | **5.38 (1.02–28.29)** | **0.047** |

Ref, reference category; OR, odds ratio; 95% CI, 95% confidence interval; Crude Model, univariate analysis; Adjusted Model, adjusted for potential confounders with a univariate analysis p < 0.100; bold values, it indicates that it is statistically significant.
