# Supplementary material for: Polymorphisms in MTNR1A (rs2119882) and CLOCK (rs1801260) genes are associated with facial acne susceptibility in gas station workers
Source: PLoS One. 2025 Jul 24;20(7):e0329150. doi: 10.1371/journal.pone.0329150 (PMC12289049; doi:10.1371/journal.pone.0329150)
Supplement: S4 Table — (DOCX) [file pone.0329150.s004.docx]

**S4 Table. Case-control analysis of the association between *MTNR1A* and *CLOCK* gene polymorphisms and acne risk using the AFG as a control.**

| ***Gene*** | **Gene model** | **Genotype** | **Crude Model** | | **Adjusted Model** | |
| --- | --- | --- | --- | --- | --- | --- |
|  |  |  | ***OR* (95% *CI*)** | ***p*-value** | ***OR* (95% *CI*)** | ***p*-value** |
| ***MTNR1A* gene rs2119882 locus** | **Codominant** | **TT** | *Ref* |  | *Ref* |  |
|  |  | **TC** | 1.49 (0.45–4.96) | 0.516 | 1.37 (0.36–5.16) | 0.644 |
|  |  | **CC** | 1.77 (0.46–6.78) | 0.406 | 1.82 (0.41–8.06) | 0.433 |
|  | **Dominant** | **TT** | *Ref* |  | *Ref* |  |
|  |  | **TC+CC** | 1.59 (0.53–4.77) | 0.407 | 1.52 (0.45–5.2) | 0.503 |
|  | **Recessive** | **TT+TC** | *Ref* |  | *Ref* |  |
|  |  | **CC** | 1.41 (0.45–4.45) | 0.560 | 1.50 (0.43–5.2) | 0.525 |
|  | **Overdominant** | **TT+CC** | *Ref* |  | *Ref* |  |
|  |  | **TC** | 1.15 (0.41–3.2) | 0.793 | 1.02 (0.34–3.07) | 0.975 |
|  | **Additive** | **-** | 1.33 (0.68–2.61) | 0.399 | 1.35 (0.64–2.84) | 0.433 |
| ***CLOCK* gene rs1801260 locus** | **Codominant** | **AA** | *Ref* |  | *Ref* |  |
|  |  | **AG** | 3.03 (0.7–13.23) | 0.140 | 5.39 (0.91–32.08) | 0.064 |
|  |  | **GG** | 3.9 (0.38–40.37) | 0.254 | 4.92 (0.42–57.72) | 0.205 |
|  | **Dominant** | **AA** | *Ref* |  | *Ref* |  |
|  |  | **AG+GG** | 3.25 (0.89–11.9) | 0.075 | **5.23 (1.16–23.53)** | **0.031** |
|  | **Recessive** | **AA+AG** | *Ref* |  | *Ref* |  |
|  |  | **GG** | 3.22 (0.32–32.89) | 0.324 | 3.89 (0.33–46.1) | 0.282 |
|  | **Overdominant** | **AA+GG** | *Ref* |  | *Ref* |  |
|  |  | **AG** | 2.74 (0.63–11.82) | 0.177 | 4.76 (0.81–28.06) | 0.085 |
|  | **Additive** | **-** | 2.35 (0.87–6.39) | 0.093 | 3.08 (0.99–9.54) | 0.051 |

Ref, reference category; OR, odds ratio; 95% CI, 95% confidence interval; Crude Model, univariate analysis; Adjusted Model, adjusted for potential confounders with a univariate analysis *p* < 0.100; bold values, it indicates that it is statistically significant.
